# Transthyretin Anti-Amyloidogenic and Fibril Disrupting Activities of *Bacopa monnieri* (L.) Wettst (Brahmi) Extract

**DOI:** 10.3390/biom9120845

**Published:** 2019-12-09

**Authors:** Fredrick Nwude Eze, Kornkanok Ingkaninan, Porntip Prapunpoj

**Affiliations:** 1 Department of Biochemistry, Faculty of Science, Prince of Songkla University, Hat Yai, Songkhla 90112, Thailand; fredrickeze@rocketmail.com; 2 Department of Pharmaceutical Chemistry and Pharmacognosy, Faculty of Pharmaceutical Sciences and Center of Excellence for Innovation in Chemistry, Naresuan University, Phitsanulok 65000, Thailand; kornkanoki@nu.ac.th

**Keywords:** antioxidants, amyloidosis, *Bacopa monnieri*, bioactive compounds, degenerative diseases, fibrillation, transthyretin

## Abstract

The homotetrameric plasma protein transthyretin (TTR), is responsible for a series of debilitating and often fatal disorders in humans known as transthyretin amyloidosis. Currently, there is no cure for TTR amyloidosis and treatment options are rare. Thus, the identification and development of effective and safe therapeutic agents remain a research imperative. The objective of this study was to determine the effectiveness of *Bacopa monnieri* extract (BME) in the modulation of TTR amyloidogenesis and disruption of preformed fibrils. Using aggregation assays and transmission electron microscopy, it was found that BME abrogated the formation of human TTR aggregates and mature fibrils but did not dis-aggregate pre-formed fibrils. Through acid-mediated and urea-mediated denaturation assays, it was revealed that BME mitigated the dissociation of folded human TTR and L55P TTR into monomers. ANS binding and glutaraldehyde cross-linking assays showed that BME binds at the thyroxine-binding site and possibly enhanced the quaternary structural stability of native TTR. Together, our results suggest that BME bioactives prevented the formation of TTR fibrils by attenuating the disassembly of tetramers into monomers. These findings open up the possibility of further exploration of BME as a potential resource of valuable anti-TTR amyloidosis therapeutic ingredients.

## 1. Introduction

Transthyretin (TTR) is one of the most abundant plasma proteins and is involved in the transport of thyroxine and vitamin A [1,2]. In the cerebrospinal fluid, this 55 kDa homotetrameric protein synthesized in the choroid plexus is the main transporter of thyroxine (T4). Recent evidence seems to suggest a neuroprotective role of TTR in Alzheimer’s disease. However, TTR in its pathological form, amyloid transthyretin (ATTR), is responsible for a spectrum of progressive, debilitating, life-altering neurodegenerative diseases known as ATTR amyloidosis which originated from the misfolding, mis-aggregation and systemic deposition of ATTR in several organs. ATTR amyloidosis is a severe clinical disorder leading to death in many cases within seven to ten years after the onset of clinical manifestations [1]. At the moment there is no cure for the disease and effective therapeutic interventions are very limited. Thus, there is a pressing need for investigations targeted toward the identification or development of safe and effective therapeutic interventions.

Native wild-type TTR is fairly stable under physiological conditions but possesses an intrinsic propensity to dissociate into its monomeric form. This propensity is enhanced by single point mutations in the TTR gene leading to variant TTR with altered and often reduced quaternary structural stability. At the moment, there are over 130 variant forms of TTR, most of which are amyloidogenic. Systemic deposition of aggregated variant TTR as amyloid fibrils in various organs of the body constitutes the pathological hallmark of hereditary variant transthyretin (ATTRv) amyloidosis, such as the formally known familial amyloidotic polyneuropathy (FAP) and familial amyloidotic cardiomyopathy (FAC). Deposition of wild-type transthyretin leads to wild-type transthyretin (ATTRwt) amyloidosis or the erstwhile senile systemic amyloidosis. ATTRwt amyloidosis is a leading cause of death in the elderly often involving heart failure [3]. While the complete picture of the molecular mechanisms underlying the transition of normal soluble functional transthyretin into its pathological counterpart is still emerging, the prevailing hypothesis is that the initial and most important step is the dissociation of the native tetramer into monomers [1]. Thus, investigations involving the identification and development of small-molecule ligands that prevented tetramer dissociation, also known as kinetic stabilizers, were considered a viable therapeutic strategy [4]. The recent development of kinetic stabilizers, such as Tafamidis and diflunisal, in addition to TTR gene silencers, including Inotersen and Patisiran, has provided patients with some positive treatment outcomes. However, limitations involving the long-term consequences and failure to prevent neurologic impairment in patients remain major sources of concern [5]. The investigations into safe and effective alternative therapeutic agents for preventing TTR amyloidogenesis and/or disrupting the preformed fibrils (amyloid disrupters), therefore, are still required. Recently, natural products from plants have been receiving increasing attention for their anti-amyloid activities as well as alternative beneficial effects, such as antioxidants, anti-inflammatory, and metal-chelating properties [6,7,8].

*Bacopa monnieri* (L.) Wettst also commonly known as Brahmi, Prom-mi, or water hyssop, is a small, perennial herb commonly found in the marshy areas of Asia and many tropical and subtropical regions around the world. *B. monnieri* is a member of the family Plantaginaceae for which there are about 100 species under the same genus. Three species of the plant are common in Thailand viz., *B. floribunda* (R. Br.) Wettst (local name: Phak sam Ian), *B. caroliniana* (Walter) B. L. Rob (local name: Lam pailin), and *B. monnieri* (L.) Wettst (local name: Prom mi). *B. monnieri* is the most common of the three due to its prevalent use in Thai traditional medicine for alleviating cognitive impairment and enhancing intelligence [9]. For thousands of years, Brahmi was widely used in Ayurveda, the Indian traditional system of medicine for treating several neurological disorders and for improving overall well-being [10]. Several pharmacological investigations have demonstrated the antioxidant [11], anti-inflammatory [12], and neuroprotective effects on disorders, such as Alzheimer’s disease, Parkinson’s disease, and brain injury [13]. However, its impact on ATTR amyloidosis has yet to be investigated. Given its reportedly good safety profile [14] and abundance of bioactive metabolites [15], the objective of the present study was thus to determine the effect of *Bacopa monnieri* extract (BME) on transthyretin amyloidogenesis and fibril disruption. Knowledge from this investigation could provide insights pertaining to the therapeutic potential of BME against ATTR amyloidosis.

## 2. Materials and Methods

### 2.1. Expression and Purification of Recombinant L55P TTR

Recombinant L55P TTR was produced in *Pichia pastoris* expression system as described earlier [16]. L55P TTR was purified from the concentrated culture supernatant using preparative discontinuous native-PAGE. Silver staining was used to determine fractions containing only L55P TTR, which were subsequently pooled and concentrated by ultrafiltration. Concentration of the purified L55P TTR was determined by Bradford assay using bovine serum albumin as standard. Pure L55P TTR was stored at −20 °C until use.

### 2.2. Purification of Human TTR from Plasma

Human plasma was pretreated by reduction of albumin via adsorption in a Cibacron blue 3GA (Sigma-Aldrich, St. Louis, MO, USA) column. The unbound faction was concentrated by ultrafiltration. Human TTR was purified from the concentrated, pretreated human plasma by preparative discontinuous native-PAGE using BIO-RAD Model 491 Prep Cell system (BIO-RAD, Hercules, CA, USA) as described previously [17].

### 2.3. Plant Material Collection and Preparation of B. monnieri Extract (BME)

Fresh Brahmi was obtained from Naresuan University. Whole plant specimen was identified and authenticated by Dr. Pranee Nangngam with voucher specimen (Saesong004) deposited at the Herbarium of the Department of Biology, Faculty of Science, Naresuan University, Thailand. Brahmi aerial parts of about 10 cm was washed and dried for 24 h at 50 °C in a hot air oven. The dried plant material was then blended into powder. Brahmi powder was extracted as earlier reported [15]. Briefly, pre-soaked plant material was extracted with 95 % ethanol (solid solvent ratio of 1:6 *w*/*v*) by sonicating for 10 min. The residue was further extracted two more times. The ethanolic extracts were combined, filtered and evaporated to obtain *Bacopa monnieri* or Brahmi extract (BME).

### 2.4. Chemical Characterization of Brahmi Extract

#### 2.4.1. RP-HPLC Quantitative Analysis

It has been widely reported that saponins constitute the major bioactive components in *B. monnieri*, thus the saponin content of Brahmi was quantified using reverse-phase high-performance liquid chromatography as previously reported [18]. Five individual saponins standards, including Bacoside A3, Bacopaside I, Bacopaside II, Bacopaside X, and Bacopopasaposin C were used. Total saponin content was obtained as the sum of the individual saponin and expressed as the percentage weight of the dried extract.

#### 2.4.2. Total Phenolic Content

The content of phenolic compounds in BME was determined by Folin–Ciocalteu as previously described [19]. BME or gallic acid (standard) was solubilized in DMSO: methanol (10:90 *v*/*v*). One hundred microliters of BME, standard or blank was added into test tube followed by freshly prepared 10 % Folin–Ciocalteu reagent (200 µL). For the blank, 200 µL of distilled water was added in place of Folin–Ciocalteu reagent. Five minutes later, 700 mM sodium carbonate solution (800 µL) was added to develop a blue mixture. The solutions were incubated in the dark for 2 h at room temperature. Absorbance was read at 765 nm and a standard curve was plotted from gallic acid (0–0.05 mg). Total phenolic content of BME was obtained from the standard curve and expressed as mg gallic acid equivalent per gram of Brahmi extract dry weight.

#### 2.4.3. Total Flavonoid Content

Content of flavonoid compounds was determined by aluminum chloride colorimetric assay as previously described [19]. One hundred and eighty microliters of BME, quercetin (standard) or blank solubilized in DMSO: methanol (1:5 *v*/*v*) was introduced to test tubes. Methanolic aluminum chloride 10% *w*/*v* (30 µL) was added into the solution. Methanol was added to the blank instead of AlCl_3_. Subsequently, 1 M sodium acetate (30 µL) and distilled water (850 µL) were added to the mixture and vortexed. Due to the deep coloration of the extract, a blank for the extract was prepared containing all the components but with methanol instead of methanolic AlCl_3_ solution. The sample, standard and blank solutions were incubated in the dark at room temperature for 30 min. Absorbance was recorded at 415 nm. A standard curve was plotted from quercetin (0–0.05 mg). The total flavonoid content of Brahmi extract was derived from the standard curve and expressed as mg quercetin equivalent per gram of Brahmi extract dry weight.

#### 2.4.4. Ferric Reducing Antioxidant Power (FRAP) Assay

The antioxidant capacity of Brahmi extract was obtained using FRAP assay as described by Benzie and Strain [20] with slight modifications. Freshly prepared FRAP solution (300 mM acetate buffer pH 3.6, 10 mM 2,4,6-Tris(2-pyridyl)-s-triazine) solution, 20 mM FeCl_3_·6H_2_O) was warmed for 30 min at 37 °C in the dark. BME was solubilized in 50% DMSO while ferrous sulfate (0–2 mM) was prepared as standard for calibration. BME or ferrous sulfate solution (10 µL) was added in a 96-well microplate followed by FRAP solution (200 µL). The mixture was incubated at 37 °C for 30 min in the dark. Absorbance was read at a wavelength of 593 nm. FRAP values were obtained from a calibration curve prepared using ferrous sulfate and expressed as mmol ferrous equivalent per gram of Brahmi extract dry weight.

#### 2.4.5. DPPH Radical Scavenging Assay

The anti-radical activity of BME was determined by DPPH assay as described by Lesjak et al. [21]. Ten microliters of plant extract solution (8.33–166.67 mg/L) was added into the wells of a 96-well microtiter plate. Methanol (140 µL) followed by 0.1 mM DPPH (150 µL) in methanol were added to the extract. Methanol without the extract served as control while corresponding blank probes contained 10 µL of extract and 290 µL of methanol. After 30 min of incubation, absorbance was read at 515 nm and the DPPH radical scavenging effect of BME was expressed as IC_50_, i.e., the extract concentration that inhibited DPPH radical formation by 50%.

### 2.5. Transthyretin Tetramer Stabilization Assays

#### 2.5.1. Urea-Mediated Denaturation Assay

The effect of BME on human TTR or L55P TTR tetramer stability was determined using urea-mediated denaturation assay. The protein was pre-incubated with BME, curcumin or DMSO (solvent) for 4 h at 37 °C in the dark. Urea solution was added to the protein solution (at a final concentration of 6 M) to initiate dissociation of the tetramers. Protein solution was incubated at 4 °C in the dark for 72 h. Thereafter, the amount of folded L55P TTR (i.e., dimers, trimmers, and tetramers) left was obtained by resolving the protein solution on 10% Tricine SDS-PAGE gel followed by densitometric analysis on LabWorks 4.0 software (UVP Ltd., Cambridge, UK) of the Coomassie brilliant blue R-250, stained protein bands on the gels.

#### 2.5.2. Acid-Mediated Denaturation Assay

Human TTR solution with or without BME was pre-incubated for 4 h to enable binding interactions. Thereafter, protein solutions were subjected to denaturation by addition of acetate buffer, pH 4.0 and incubated at 37 °C in the dark for 14 days. To determine the extent of denaturation, protein solutions were solubilized in SDS sample loading buffer without β-mercaptoethanol (i.e., 0.05 M Tris-HCl, pH 6.8, 10% glycerol, 2% SDS, and 0.1% bromophenol blue dye). Protein samples (without boiling) were resolved on 12% polyacrylamide gel containing 0.1% SDS and separated bands were detected with Coomassie brilliant blue R-250 staining. In principle, under this SDS-PAGE condition, tetrameric transthyretin will be observed as SDS-resistant dimers on the gel [22]. The fraction of human TTR tetramers left after subjection to mild acid denaturation stress was quantified from the gel band intensities via densitometric analysis using gel documentation.

### 2.6. Transthyretin pH-Induced Fibril Formation and Disruption Assays

The effect of BME on human TTR aggregation and fibril formation was determined by pre-incubating human TTR for 4 h with or without BME at 37 °C. Thereafter, the pH was adjusted to 4.0 with 200 mM acetate buffer, pH 4.0. Aggregation of human TTR was enabled by aging the protein complex for 14 days in the dark at 37 °C under aseptic conditions. The extent of protein aggregation and fibrillation were determined by transmission electron microscopy (TEM). For TEM analysis, a drop of protein solution was spotted unto a formvar-coated copper grid for 3 min. Excess fluid was carefully blotted with a filter paper wedge. The grid was then rinsed with a drop of Milli-Q water (Millipore, Billerica, MA, USA) followed by staining with 2% uranyl acetate in 70% methanol for 2 min. The grid was further rinsed with a drop of Milli-Q, excess fluid blotted away and dried at ambient temperature. The TEM image was obtained using a JEOL JEM-2010 (JEOL Ltd., Akishima, Tokyo, Japan) electron microscope operating at 160 kV. The fibril disruption assay was performed as previously described [6]. Preformed fibrils were prepared using acid-mediated aggregation assay and formation of fibrils was confirmed by TEM. The pre-formed fibrils were further incubated with or without BME in the dark at 37 °C for 24 h. Thereafter, fibril disruption activity was confirmed by TEM and glutaraldehyde cross-linking assay followed by 10% Tricine-SDS-PAGE.

### 2.7. Transthyretin Quaternary Structural Alterations Determined by Glutaraldehyde Cross-Linking Assay

The quaternary structure of human TTR or L55P TTR subjected to urea-induced denaturation or human TTR after acid-mediated denaturation stress was determined by chemical cross-linking assay using glutaraldehyde. Aliquot of protein solution was obtained denaturation at stress. Glutaraldehyde was added to the protein solution (final concentration of 2.5%) and cross-linking allowed for 4 min at ambient temperature. Cross-linking reaction was quenched by addition of 7% NaBH_4_ in 0.1 M NaOH (of equal volume to glutaraldehyde). The mixture was solubilized with 4× SDS sample loading buffer (final SDS concentration of 2%) and boiled for 10 min before resolving on 10% Tricine-SDS-PAGE gel. Protein bands were detected using Coomassie brilliant blue staining.

### 2.8. Determination of Inhibitor Binding by ANS Displacement Fluorescence Assay

The small fluorescent probe 8-anilino-1-naphthalene sulfonic acid (ANS), is widely used in monitoring ligand or inhibitor binding to the thyroxine-binding site of native tetrameric TTR [23]. ANS binding assay was performed on Synergy HT (BioTek Instruments, Winooski, VT, USA) microplate reader. The ANS stock solution (2.47 mM) was prepared in 10 mM phosphate buffer, pH 7.4 and its concentration was determined by absorbance (E_350_ = 5000 M^−1^cm^−1^) [24]. Human TTR (0.055 µg/µL) was incubated in the presence of 10 µM ANS and varying concentrations of BME (0.0055–0.55 µg/µL). Fluorescence intensity was measured after 10 min at excitation of 360/40 nm and emission at 460/40 nm at a temperature of 37 °C.

### 2.9. Statistical Analysis

Statistical analysis was performed with the aid of Graph Pad Prism version 7 for Microsoft windows (Graph Pad Software, San Diego CA, USA). The data was statistically analyzed by one-way analysis of variance (ANOVA) followed by multiple comparison analysis using Tukey’s test. Statistical significance was defined as * *p* < 0.05, ** *p* < 0.01, and *** *p* < 0.001. All determinations were performed in triplicate and results represented as means ± SD.

## 3. Results

### 3.1. BME Prevented TTR Amyloid Fibril Formation

**Figure 1:**
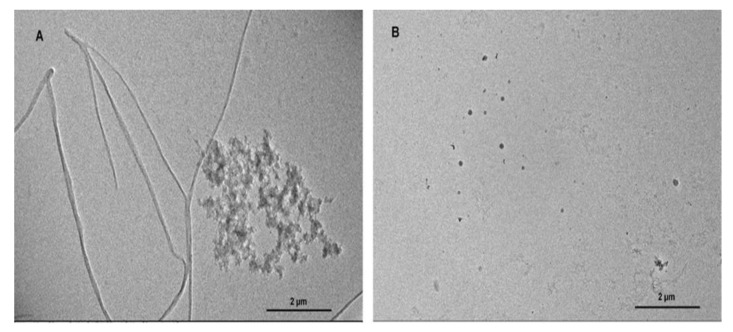
The effect of BME on human TTR aggregation and fibril formation. TEM images of human TTR subjected to acid-mediated aggregation assay in the absence (**A**) or presence (**B**) of BME after 14 days of incubation at 37 °C.

Amyloidogenesis and fibril formation by TTR is closely associated with the development of the devastating clinical features that characterize ATTR amyloidosis. In order to determine whether BME is able to modulate TTR amyloidogenesis, we accessed its effect on human TTR fibrillation in vitro using acid-mediated aggregation and fibril formation assay. In vitro, human TTR is capable of forming mature amyloid fibrils under mildly acidic conditions at a temperature of 37 °C. As revealed by the TEM image in Figure 1A, human TTR did not only form amorphous aggregates, but also formed mature amyloid fibrils after incubation for 14 days in the absence of BME (Figure 1B). Notably, in the presence of 20 µg/µL BME (concentration of human TTR was 1 µg/µL) no oligomers nor fibrils were observed as shown in Figure 1B. From these results, it can be inferred that BME inhibited human TTR aggregation and fibril formation.

### 3.2. Effect of Native TTR Stability under Urea-Induced Denaturation Stress

**Figure 2:**
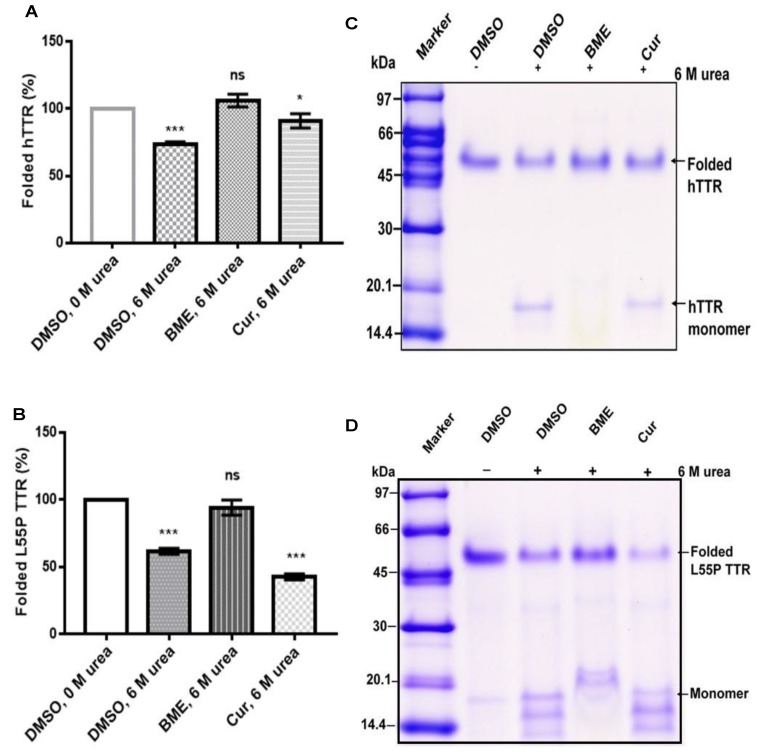
The effect of BME on native TTR dissociation under urea mediated denaturation stress. Bar charts (**A**,**B**) represent folded human TTR (hTTR) and L55P TTR, respectively, in percentage left after 72 h of incubation in the presence or absence of BME. Images (**C**,**D**) are representative of Tricine-SDS-PAGE images of human TTR and L55P TTR, respectively, after denaturation.

Since tetramer dissociation into monomers is the required and rate-limiting step in TTR amyloidogenesis we speculated that BME might have an effect on native TTR dissociation given its inhibition of fibril formation. Thus, we determined the effect of BME on human TTR and L55P TTR resistance to urea-mediated denaturation stress as described in the Materials section. As shown in Figure 2A (and its representative Tricine-SDS-PAGE image, Figure 2C), in the absence, but not in the presence of BME, human TTR was substantially dissociated after 72 h of incubation in 6 M urea at 4 °C. The percent of folded human TTR (i.e., tetramers, trimers and dimers) left after denaturation stress was higher in the presence of 13.33 µg/µL BME (106 ± 4.77) compared to in its absence (73.49 ± 2.06). Quantitatively the percentage of folded human TTR left after denaturation in BME was not different from that of the control. Correspondingly, there was no formation of human TTR monomers in the presence but not in the absence of BME as indicated by the lack of appearance of the 16 kDa protein band on the gel (Figure 2C). The same trend of results was observed when L55P TTR was subjected to urea mediated denaturation with or without 13.33 µg/µL BME (Figure 2B,C), however, dissociation of L55P TTR was not completely abrogated by BME as indicated by the presence of protein bands around 14–20 kDa corresponding to monomers. These results thus suggest that BME mitigated the dissociation of both human TTR and L55P TTR into monomers.

### 3.3. Effect of Human TTR Tetramer Stability under Mildly Acidic Denaturation Condition

**Figure 3:**
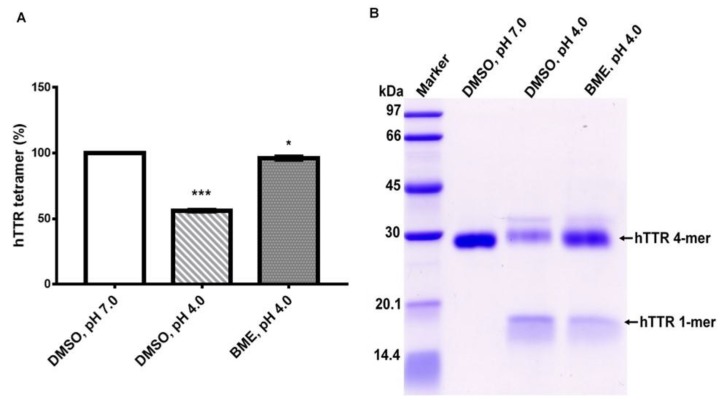
(**A**) Quantitative analysis of the effect of BME on human TTR (hTTR) tetrameric resistance to moderate acidic denaturation conditions. (**B**) Representative Tricine-SDS-PAGE gel image of human TTR (hTTR) resistance to acid mediated denaturation without (Lane 3) or with (Lane 4) BME. Lane 1—protein molecular weight marker.

TTR undergoes amyloidogenesis in vitro under mildly acid pH. However, this process requires the dissociation of native TTR tetramers into monomers which are susceptible to amyloidogenic transformation by partial unfolding. To ascertain the effect of BME on human TTR stability under acid-mediated denaturation conditions, the amount of tetramer left after aging the protein for 14 days with or without BME was quantified as described in above. As shown in Figure 3, incubation of human TTR under mild acidity markedly reduced the percentage of tetramers in the absence but not in the presence of 20 µg/µL BME. Correspondingly, the content of monomers produced due to acid mediated denaturation of human TTR was higher in the absence than in the presence of BME as revealed by the monomer band intensity (Figure 3B). These results suggest that BME mitigated human TTR tetramer dissociation under mildly acidic conditions and further supports the earlier results observed under urea mediated denaturation conditions.

### 3.4. Impact of BME on TTR Quaternary Structural Stability and Dose-Dependent Effect

**Figure 4:**
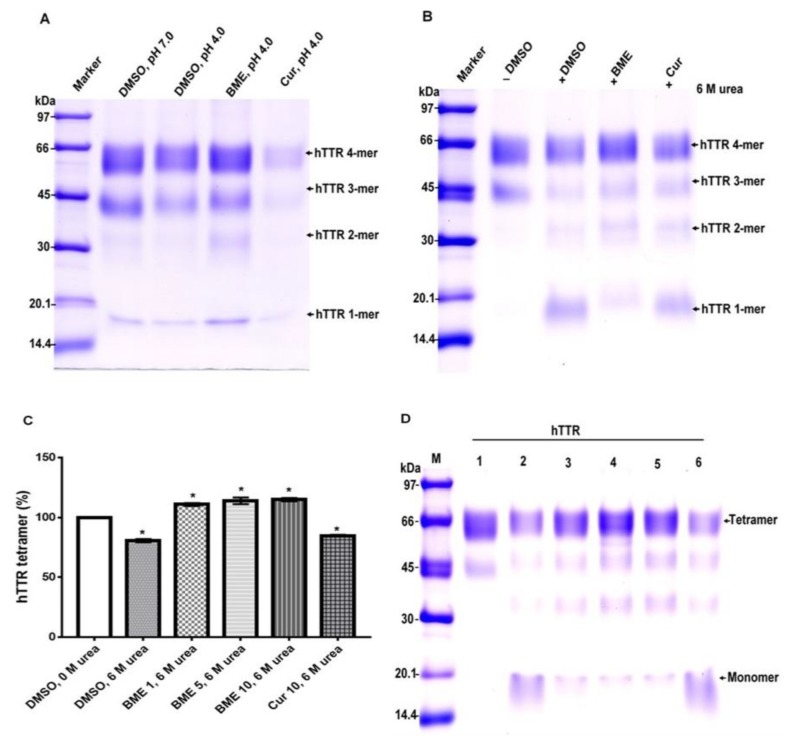
The effect of BME on human TTR (hTTR) quaternary structural stability and its dose-dependent effect. Representative Tricine-SDS-PAGE gel images of glutaraldehyde cross-linked protein samples with or without BME after acid-mediated denaturation (**A**) and urea-mediated denaturation (**B**) assays. (**C**) Quantitative analysis of dose-dependent activity of BME on urea-mediated denaturation of human TTR. (**D**) Representative Tricine-SDS-PAGE gel image of the effect of increasing BME amounts on human TTR tetramer stability. M—molecular weight marker; 1—human TTR in DMSO, without urea; 2—human TTR in DMSO, 6M urea; 3—human TTR in 1 µg/µL BME, 6M urea; 4—human TTR in 5µg/µL BME, 6M urea; 5—human TTR in 10µg/µL BME, 6M urea; 6—10µg/µL curcumin, 6M urea.

As earlier stated, the key to TTR amyloidogenesis is its transition from tetramer to monomer, thus the stability of the native tetramer is crucial in mitigating its amyloidogenesis. To determine the effect of BME on the quaternary structural stability of human TTR, the protein solution with or without the extract was subjected to urea mediated denaturation stress followed by cross-linking with glutaraldehyde. Glutaraldehyde cross-linking assay gives a reflection of the protein in its different conformations upon resolving on Tricine-SDS-PAGE [25]. As shown in Figure 4, the human TTR solution with or without BME after denaturation stress contained tetramers, trimers, dimers, and monomers, albeit in different proportions. In both the acid and urea mediated denaturation conditions (Figure 4A,B, respectively), the proportion of human TTR tetramer left at the end of the denaturation stress was greater in the presence than in the absence of BME as indicated by the protein band intensities. The opposite was reflected with regards to the proportion of monomers left, especially in the case of urea mediated denaturation (Figure 4B). However, based on the protein band intensities, the content of monomers left after acid mediated denaturation appear to be lesser in the absence of BME. Presumably, this may be due to their rapid conversion to amyloid competent species that are rapidly transformed into higher molecular weight aggregates and fibrils [26] as earlier shown in Figure 1. Thus, it is possible to deduce from these results that BME resisted the dissociation of TTR tetramer to monomers under tetramer-dissociating acid and urea mediated denaturation stress conditions.

The dose-dependent effect of BME on human TTR stability was determined by varying the concentrations of the extract in human TTR denaturation solution. As shown in Figure 4C, there was a substantially higher percentage of human TTR tetramers left after denaturation stress in the presence and not the absence of BME. However, the increase in the amount of human TTR in the presence of increasing amounts of BME was not remarkably different. These observations may be due to fact that the bioactive ligands responsible for tetramer stability was already saturated in its interactions with human TTR at every concentration of BME tested. These results thus seem to suggest that it is possible for BME to stabilize human TTR tetramer.

### 3.5. Determination of Human TTR Amyloid Fibril Disrupting Activity of BME

**Figure 5:**
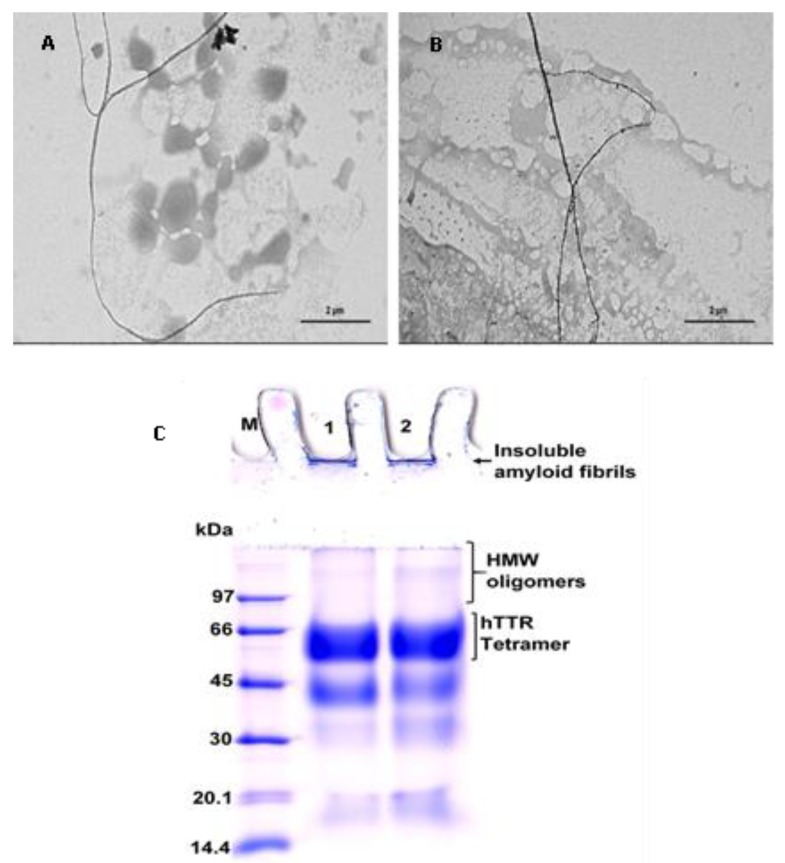
Images of pre-formed human TTR amyloid fibrils in the absence (**A**) or presence of BME (**B**) after incubation for 24 h. (**C**) Tricine-SDS-PAGE gel representation of human TTR fibril disruption activity of BME. Lane 1—protein molecular weight marker; Lane 2—human TTR pre-formed fibrils without BME; Lane 3—human TTR pre-formed fibrils with BME.

In order to ascertain whether BME had any effect on already formed human TTR amyloid fibril, the soluble protein was first converted into mature fibrils by aging for 14 days in 200 mM acetate buffer, pH 4.0 at 37 °C. Formation of mature human TTR amyloid fibrils was confirmed using TEM. The preformed human TTR fibrils was the co-incubated with or without BME at 37 °C for an additional 24 h. Thereafter, aliquot of the fibril solution was obtained and diluted by 100 times with Milli-Q water and spotted on formvar coated copper TEM grid followed by negative staining with 2% uranyl acetate. Electron micrographs were later obtained. From the TEM images obtained (Figure 5), it could be seen that the presence of BME did not alter the fibril morphology. There was no disruption in the mature preformed human TTR fibrils after 24 h of incubation of the given concentration of BME. Thus, it can be concluded that BME did not possess any fibril disrupting activity on mature human TTR fibrils.

### 3.6. Determination of BME Binding to TTR

**Figure 6:**
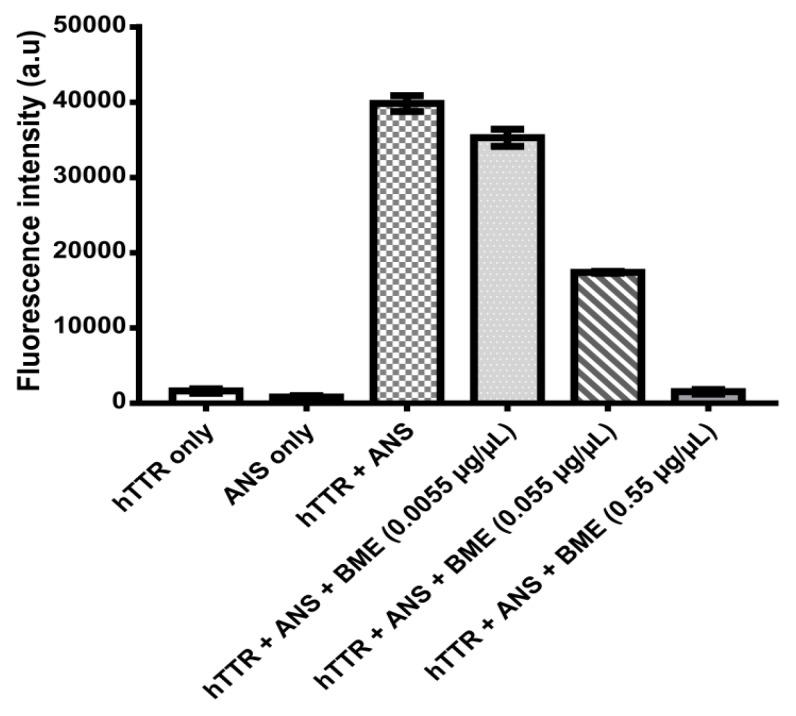
Human TTR-ANS fluorescence intensity without or with increasing concentrations of BME for the determination of ANS displacement from human TTR T4-binding sites by BME.

Binding interaction between BME and human TTR was monitored using the small fluorescent probe ANS. ANS is very sensitive to the polarity of its environment. In aqueous environments, ANS has a weak fluorescence and displays an emission of maximum at 500 nm. Upon binding to the hydrophobic patches of proteins it produces a blue shift in its emission wavelength and an increase in its fluorescence intensity the extent of which is dependent on the protein structure and milieu surrounding ANS [27]. In the presence of human TTR tetramer, ANS binds specifically to the thyroxine-binding sites within the hydrophobic cavity with a consequent increase in fluorescence intensity at about 480 nm [28]. As shown in Figure 6, human TTR and ANS separately in phosphate buffer had very weak fluorescence intensities. However, upon co-incubation of human TTR and ANS in phosphate buffer, there was a dramatic increase in the fluorescence intensity. This can be attributed to the binding of ANS to the thyroxine-binding sites of human TTR tetramers as previously reported [7,28]. Interestingly, introduction of BME reduced the ANS fluorescence intensities dose-responsively. These seem to suggest that BME binds to the thyroxine binding sites of the tetramers and prevents ANS from its preferred binding location on the protein. Out of the hydrophobic T4-binding sites, ANS thus gives reduced fluorescence intensity.

### 3.7. Chemical Characterization of BME

The major bioactive phytoconstituents in BME were determined by RP-HPC quantitative analysis as described in the Methods section. BME was found to contain Bacoside A (Bacoside A3 (2.22% *w*/*w*), Bacopaside II (4.68% *w*/*w*), Bacopaside X (3.25% *w*/*w*)) and Bacopaside I (3.54% *w*/*w*), giving a total saponin content of 16.03% *w*/*w* of dried extract. Previously it had been reported that the total Bacoside A content of methanol extract of in vitro culture of *Bacopa monnieri* was 8.73 mg/g of dry weight [29]. The superior content of BME may be due to difference in our extraction method and solvent composition. BME contain over sixty individual compounds. The metabolite profile of saponins, phenolics, and other components present were previously determined by LC-ESI-qTOF-MS [15]. The total phenolic content of BME was also determined using the widely accepted Folin–Ciocalteu assay. BME recorded a phenolic content of 26.45 mg gallic acid equivalent/gram of dry weight. Compared to the phenolic content of thirty common culinary herbs in Thailand previously reported, BME had a higher phenolic content than twenty-three of them including *Cucurbita moschata* (pumpkin) leaves, *Allium sativum* (garlic) cloves, *Mentha canalenisa* (peppermint) leaves, *Curcuma longa* (turmeric) rhizome. However, the phenolic content of *Ocimum basilicum* (sweet basil) leaves, *Ocimum sanctum* (holy basil) leaves and *Acacia pennet*a (acacia) shoot tips were higher than BME. Given that flavonoids constitute a major proportion of polyphenols in several plant species, the total flavonoid content in BME was determined and found to be 17.6 mg quercetin equivalent/g of BME [30].

The antioxidant capacity of BME was determined using DPPH and FRAP assays, two of the most commonly reported in vitro methods for ascertaining antioxidant content in fruits, vegetables, and plant-derived constituents. The FRAP assay measures the reductive ability of all the redox-active constituents in BME via single electron transfer (SET) mechanism while the DPPH assay determines radical scavenging activity of the extract toward the DPPH radical by SET and hydrogen atom transfer (HAT) mechanisms [31]. As shown in Table 1, BME had a FRAP value of 306.82 µmol Fe(II)/g, which is indicative of its ability to reduce ferric-TPTZ complex to its ferrous form. The DPPH IC_50_ value for BME was 90.89 mg/L. Konczak et al. observed a high degree of correlation between the FRAP value (in µmol Fe(II)/g dry weight) of commercially grown native Australian herbs and spices and their phenolic content (in mg gallic acid equivalent/g dry weight), which validated the notion that antioxidant capacity of bioactive components from food and plants is a function of their natural phenolic content [32]. Given that many of the health benefits associated with the consumption of fruits, vegetables and bioactives from plant sources have been attributed to antioxidants, it can be speculated that the presence these small-molecule natural antioxidant phenolics and saponins might be responsible for the anti-amyloidogenic properties of BME.

## 4. Discussion

The objective of this work was to determine the ability of *Bacopa monnieri* extract (BME) to modulate transthyretin amyloidogenesis and fibril disaggregation in vitro. The results obtained strongly suggest that BME inhibited the formation of human TTR aggregates and mature fibril by stabilizing and preventing the dissociation of native tetramers through binding at the thyroxine-binding sites of the protein. However, BME could not disrupt preformed TTR fibrils. These findings suggest that BME contained bioactive metabolites with potential therapeutic implications for the modulation of ATTR amyloidosis.

Normal physiological native TTR is made up of four identical monomers often annotated A-D. Each monomer is consisted of eight B-strands (A–H) and a short alpha helix, EF helix. Interaction between monomers via hydrogen bonds produces dimers. The dimers self-associate furnished by hydrogen bonds and hydrophobic interactions to form the functional tetramer which possesses a central hydrophobic channel that contains two binding sites for thyroxine (T4) created by amino acid residues from both dimers [33]. TTR is also capable of acquiring a gain-in-toxic function by becoming amyloidogenic in human [34]. Indeed, TTR amyloidogenesis is associated with the development of severe clinical complications including peripheral neuropathy, autonomic dysfunctions, cardiomyopathy, or death in some cases, a condition which is generally referred to as ATTR amyloidosis. Unlike in several other similar multifactorial diseases such as Alzheimer’s disease and Parkinson’s disease where there are a lot of controversies surrounding their origin, in ATTR amyloidosis the consensus is that the key molecular event underpinning its etiopathogenesis is destabilization of the homotetrameric structure leading to its dissociation to monomers [1]. Given the pivotal role of tetramer dissociation in initiating the TTR amyloid cascade, there had been a strong momentum in research focused on the identification and development of kinetic stabilizers i.e., small-ligand agents which binds to (typically at the T4-binding sites) and prevents dissociation of tetrameric transthyretin [4]. The kinetic stabilization strategy led to the development of the regulatory approved drug Tafamidis, for the treatment of early-stage FAP [1] or cardiomyopathy of ATTRw amyloidosis. However, with limitations in Tafamidis such as its inability to prevent progression in neuropathy as well as its lack of effectiveness in late-stage TTR amyloidosis and nonV30M ATTRv amyloidosis [35], it is, therefore, vital to continue investigations aimed at identifying and developing effective and safe therapeutic alternatives.

In vitro, transthyretin is known to form amyloid fibrils under mildly acidic pH (5–4) at physiological temperature [36]. Using the well-established acid-mediated aggregation and fibril formation assay, we found that BME completely abrogated the formation of oligomers and mature amyloid fibrils by human TTR after 14 days of incubation as shown by the electron micrographs of the aged protein solutions. These findings are supported by earlier studies demonstrating the anti-amyloidogenic potential of BME on α-Synuclein [37]. Parkinson’s disease is recently being considered as a type of amyloidosis—of which the main neuropathological diagnostic feature is the accumulation of cross-β sheet-rich aggregates of α-Synuclein as Lewy bodies in the brain [38]. The crucial step in the pathogenesis of Parkinson’s disease is the aggregation of α-Synuclein [39], which is reportedly attenuated by *B. monnieri* extract [37]. However, unlike α-Synuclein and beta-amyloid which aggregate and form fibrils via a nucleation-dependent polymerization mechanism, TTR follows a downhill polymerization of amyloid competent monomers [40]. The implication here is that formation of these amyloidogenic monomers mandates the dissociation of tetramers—the critical step in TTR amyloidogenesis. Additionally, since this process is concentration-driven, a viable strategy to prevent the TTR amyloidogenesis and fibrillation would certainly involve preserving the quaternary structural integrity of tetramers [41]. Results from this study shows that BME indeed mitigated the urea-induced dissociation of human TTR and its most pathogenic variant L55P TTR, into their constituent monomers. These results were further supported by the resistance of human TTR in the presence but not in the absence of BME against acid-mediated denaturation and fibril formation. However, BME was incapable of disrupting preformed mature human TTR amyloid fibrils. These seem to suggest that the inhibition of human TTR fibril formation by BME was due to the prevention of the rate-limiting tetramer dissociation. Previous studies in the recent past have shown that natural products such as curcumin from *Curcuma longa* (Turmeric), propolis from honeybee were able to prevent the dissociation of TTR tetramers and, thus, modulate its amyloidogenesis [7,28]. The prevailing molecular mechanism, especially in the case of propolis involved the binding of the small-molecule ligand at the T4-binding site within the central hydrophobic channel of TTR tetramer with consequent increase in the tetramer stability. BME binds to and displaced the fluorescent probe ANS from the T4-binding sites in native tetrameric TTR as revealed by ANS displacement assay. The binding of BME at the T4-binding sites presumably creates further interactions between the dimers which might explain the plausible increase in stability of TTR tetramer observed in the glutaraldehyde cross-linking assay. This is supported by similar observations in several previous studies, i.e., increased TTR tetramer stability by binding of small-ligands at the T4-binding sites [7,19,28,42], as well as by mutations which fill the T4-binding cavity [3].

BME possessed a high content of triterpenoid saponins as revealed by the RP-HPLC quantitative analysis, as well as phenolics, especially flavonoids. These findings were in accord with previous reports on the phytochemical constituents of *B. monnieri* extracts [15]. It is plausible that the tetramer stabilizing and anti-amyloidogenic effects of BME are due to the binding interactions mediated by these small-molecule ligands. In addition, BME also demonstrated a strong radical scavenging and antioxidant capacity. The radical scavenging effect of plant bioactives rich in phenolics is based on their ability to initiate single electro transfers, donate hydrogen atoms, or chelate transition metals [31]. In addition to BME binding directly and stabilizing TTR tetramers, its antioxidant bioactives might also be relevant in modulating TTR amyloidogenesis. For instance, it has been well reported that, similar to beta-amyloid in Alzheimer’s disease and α-Synuclein in Parkinson’s disease, TTR amyloidogenesis is also influenced by environmental or physiological factors, such as post-translational modifications [41]. This is supported by the fact that aging is the most relevant risk factor in these pathologies, and aging-associated oxidative modifications have been shown to modulate transthyretin amyloidogenesis and disease onset [43]. The free thiol group on Cys-10 residue has been shown to be one of the most susceptible free thiols in human plasma to oxidative modifications. Given its unique location close to the surface of the tetramer and near the thyroxine binding site, oxidative modifications of Cys-10 alters the structure of the homotetramer and reduces its stability [44]. The antioxidant and radical scavenging attributes of BME might provide an indirect protection on TTR by altering the redox potential of the milieu. A similar positive impact on TTR amyloidogenesis by natural agents with potent antioxidant activities had been previously observed in carvedilol, TUDCA, curcumin, and green tea extracts, which were shown to significantly reduce deposition of amyloid TTR despite their less impressive effect on tetramer stability [45,46,47,48]. Previous studies have reported the neuroprotective effects of *B. monnieri* in diseases such as Alzheimer’s disease [49], Parkinson’s disease [37], and neurotoxicant-induced brain dysfunction [50]. Our investigation for the first time provides in vitro evidence for the protective role of BME in modulating transthyretin amyloidogenesis.

## 5. Conclusions

In conclusion, this mechanistic investigation using biochemical assays suggested that BME inhibited TTR amyloidogenesis by attenuating the rate-limiting step in TTR amyloidogenesis pathway, that is the dissociation homotetramers into monomeric constituents by binding at the thyroxine-binding sites of and possibly stabilizing the native tetramer. Although these studies unveiled the potential of BME in countering the causative mechanism of ATTR pathology, further investigations are warranted, such as determining its potency and safety profile in disease animal models.

## Figures and Tables

**Table 1:** Chemical profile and antioxidant activity of Brahmi extract.

Chemical Property	Brahmi Extract
Total saponin content (% *w*/*w* of dried extract)	16.03 ± 0.01
Total phenolic content (mg gallic acid/g BME)	26.45 ± 0.34
Total flavonoid content (mg quercetin/g BME)	17.6 ± 1.5
Ferric reducing antioxidant power (µmol Fe (II)/g BME)	306.82
DPPH radical scavenging activity IC_50_ (mg/L)	90.84 ± 0.44
